# Entropy Generation in Cu-Al_2_O_3_-H_2_O Hybrid Nanofluid Flow over a Curved Surface with Thermal Dissipation

**DOI:** 10.3390/e21100941

**Published:** 2019-09-26

**Authors:** Muhammad Idrees Afridi, Tawfeeq Abdullah Alkanhal, Muhammad Qasim, Iskander Tlili

**Affiliations:** 1 Department of Mathematics, COMSATS University Islamabad (CUI), Park Road, Tarlai Kalan, Islamabad 455000, Pakistan; idreesafridi313@gmail.com (M.I.A.); mq_qau@yahoo.com (M.Q.); 2 Department of Mechatronics and System Engineering, College of Engineering, Majmaah University, Al-Majmaah 11952, Saudi Arabia; t.alkanhal@mu.edu.sa; 3 Department for Management of Science and Technology Development, Ton Duc Thang University, Ho Chi Minh City 758307, Vietnam; 4 Faculty of Applied Sciences, Ton Duc Thang University, Ho Chi Minh City 758307, Vietnam

**Keywords:** entropy generation minimization (EGM), hybrid nanofluid, heat transfer, energy dissipation, curved sheet, Runge-Kutta-Fehlberg method (FRKM)

## Abstract

Heat transfer and entropy generation in a hybrid nanoliquid flow caused by an elastic curved surface is investigated in the present article. To examine the effects of frictional heating on entropy generation, the energy dissipation function is included in the energy equation. The Tiwari and Dass model for nanofluid is used by taking water as a base fluid. A new class of nanofluid (hybrid nanofluid) with two kinds of nanoparticles, Copper (Cu) and Aluminum oxide (Al_2_O_3_), is considered. Curvilinear coordinates are used in the mathematical formulation due to the curved nature of the solid boundary. By utilizing similarity transformations, the modelled partial differential equations are converted into ordinary differential equations. Shooting and the Runge-Kutta-Fehlberg method (FRKM) have been used to solve the transformed set of non-linear differential equations. The expression for entropy generation is derived in curvilinear coordinates and computed by using the numerical results obtained from dimensionless momentum and energy equations. Comparisons of our numerical results and those published in the previous literature demonstrate excellent agreements, validating our numerical simulation. In addition, we have also conducted parametric studies and find that entropy generation and temperature suppress with increasing values of dimensionless radius of curvature. Furthermore, it is found that less entropy is generated in regular nanofluid as compare to hybrid nanofluid. To examine the influences of a set of embedding physical parameters on quantities of interest, different graphs are plotted and discussed.

## 1. Introduction

The efficiency of a thermal system reduces due to entropy generation. The mathematical expression for the entropy generation due to heat transfer and viscous dissipation was derived by Bejan [1]. After the substantial work of Bejan [1], the second law analysis of fluid flow under different circumstances became a very attractive research topic for theoretical the thermal scientists. Recently, Butt et al. [2] reported the second law analysis of fluid flow over an inclined stretching cylinder. The rate of entropy production in third-grade fluid was studied by Rashidi et al. [3]. The effects of non-uniform magnetic field on entropy generation are analyzed by Sheikholeslami et al. [4]. The entropy generation in a turbulent flow inside a horizontal tube is investigated numerically by Rashidi et al. [5]. Rashidi et al. [6] analyzed the effects of magnetic force on entropy generation in a rotating nanofluid flow. Makinde [7] presented the results of variable fluid properties and thermal radiation on entropy generation with Newtonian heating. The mixed convection effects on entropy generation in a nanofluid flow with mass suction/injection are studied by Makinde and Tshehla [8]. The entropy generation in a Cu-H_2_O nanofluid flow induced by a stretching disk is studied by Butt et al. [9]. Afridi et al. [10] studied the non-linear radiation effects on entropy generation in a Newtonian fluid flow. Entropy generation in a fluid flow inside a wavy cavity with porous medium is reported by Alsabery [11].

Viscous dissipation is a substantial factor in enhancing the temperature of the fluid; this is due to the conversion of mechanical energy into thermal energy. The reason is the dissipative frictional forces between the layers of viscous fluids. Gebhart [12] for the very first time studied the thermal dissipation in the natural convection flow of viscous fluid. It is a well-known fact that the velocity gradients are high in boundary layer flows [13]. Furthermore, the energy dissipation function is directly related to the velocity gradients. Therefore, it is more practical to consider viscous dissipation in the thermal analysis of boundary layer flows. Heat transfer analysis by taking into account the effects of viscous dissipation in the fluid flow of viscoelastic fluid is reported by Hsiao [14]. The influences of temperature-dependent thermal conductivity on heat transfer in a pseudo-plastic nanofluid with thermal dissipation is studied by Lin et al. [15]. Yazdi et al. [16] numerical studied the combined slip and viscous dissipation effects on the flow of power-law fluid. Hsiao [17] analyzed the viscous dissipation effects on heat transfer in the unsteady flow of viscoelastic fluid over a vertical stretching surface. Sreenivasulu et al. [18] studied the combined effects of Joule and viscous heating in the flow of viscous fluid in the presence of linear thermal radiation. Makinde [19] investigated the effects of Newtonian and viscous heating on the Sakiadis flow of nanofluid. Hsiao [20] reported the influences of thermal radiation and viscous dissipation on heat transfer in a flow of Maxwell fluid.

Nanofluid, pioneered by Choi [20], who discovered its significant increase in heat-transfer properties compared to conventional engineered fluid, has been found to serve in a wide range of engineering applications, for example, porous materials [21], fuel-cell industry [22], petroleum engineering [23] and coolants for devices. Resent, Hashim et al. [24] studied the flow of Al_2_O_3_ water nanofluid and heat transfer analysis by utilizing Buongiorno’s two-phase model. The flow of nanofluid inside a square cavity containing a circular cylinder by utilizing a two-phase nanofluid model is investigated by Alsabery et al. [25]. The effects of mixed convection on a flow of copper-water nanofluid inside a ventilated wavy cavity is reported by Boulahia et al. [26]. A comparative study of viscous fluid and nanofluid flow over a wavy surface is reported by Shenoy et al. [27]. The effects of viscous dissipation along with ohmic heating in a flow of Carreau nanofluid is studied by Hsiao [28]. The next level of nanofluid, called hybrid nanofluid, is introduced here. Hybrid nanofluid is engineered by the suspension of two different nanoparticles in a single regular heat transfer fluid. The thermal conductivity of hybrid nanofluid is superior to both regular nanofluid and working fluid under the same conditions. This amazing enhancement in the thermal conductivity of working fluid due to the suspension of two nanoparticles at a time attracts researchers to analyze the flow of hybrid nanofluids under different circumstances. For example, the mass suction effects with a magnetic field are reported by Devi and Devi [29]. Devi and Devi also studied the three-dimensional flow of hybrid nanofluids. The recent precious studies on the thermophysical properties on hybrid nanofluids are reported by Gorla et al. [30], Afridi et al. [31], Chamkha et al. [32] and Afridi et al. [33].

The primary concern of the recent study is to analyze the effects of viscous dissipation and curvature parameters on heat transfer and the rate of entropy production inside the boundary layer flow over a curved stretching surface. Aluminium oxide and silver nanoparticles are assumed to be dispersed homogeneously in the base fluid (water). The modelled flow equations are converted into dimensionless form by similarity transformations and solved numerically by using the Runge-Kutta-Fehlberg method (FRKM). To study the influences of various emerging flow parameters on flow, heat transfer and entropy generation, the obtained numerical solutions are plotted and discussed physically in detail.

## 2. Model Formulation 

**Figure 1:**
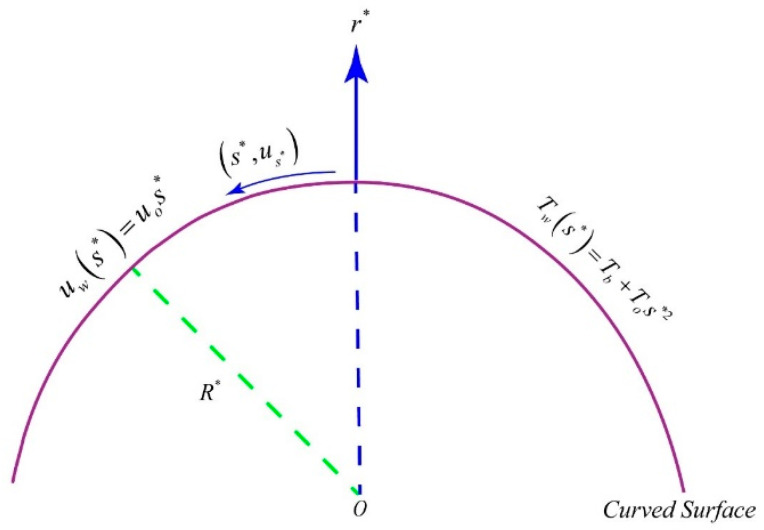
Geometry of the problem and coordinates system.

We considered a curved surface of radius $R^{*}$ and a flow of dissipative hybrid nanoliquid driven by a stretched curved surface. The curvilinear coordinate system $\left(s^{*},r^{*}\right)$ is chosen such that the $r^{*}-axis$ is normal to the boundary of the curved surface and the $s^{*}-axis$ is tangent to the surface of the curved boundary as depicted in Figure 1. The velocity and temperature profile of the stretching boundary are $u_{w}\left(s^{*}\right)=u_{o}s^{*}$ and $T_{w}\left(s^{*}\right)=T_{b}+T_{o}s^{*2}$, respectively. By taking all the assumptions we made along with the Prandtl boundary layer approximations, the equations governing the flow and heat transfer in a dissipative hybrid nanofluid take the following forms:

(1) $$\frac{\partial }{\partial r^{*}}\left(\left(r^{*}+R^{*}\right)u_{r^{*}}\right)+R^{*}\frac{\partial u_{s^{*}}}{\partial s^{*}}=0,$$

(2) $$\frac{u_{s^{*}}^{2}}{r^{*}+R^{*}}=\frac{1}{\rho_{hnl}}\frac{\partial p}{\partial r^{*}},$$

(3) $$\rho_{hnl}\left(u_{r^{*}}\frac{\partial u_{s^{*}}}{\partial r^{*}}+\frac{Ru_{s}}{r^{*}+R^{*}}\frac{\partial u_{s^{*}}}{\partial s^{*}}+\frac{u_{s^{*}}u_{r^{*}}}{r^{*}+R^{*}}\right)=-\frac{R^{*}}{R^{*}+r^{*}}\frac{\partial p}{\partial s^{*}}+\mu_{hnl}\left(\frac{\partial^{2}u_{s^{*}}}{\partial r^{*}{}^{2}}+\frac{1}{r^{*}+R^{*}}\frac{\partial u_{s^{*}}}{\partial r^{*}}-\frac{u_{s^{*}}}{{\left(r^{*}+R^{*}\right)}^{2}}\right),$$

(4) $$u_{r^{*}}\frac{\partial T}{\partial r^{*}}+\frac{R^{*}}{r^{*}+R^{*}}u_{s^{*}}\frac{\partial T}{\partial s^{*}}=\frac{k_{hnl}}{{\left(\rho c_{p}\right)}_{hnl}\left(r^{*}+R^{*}\right)}\frac{\partial }{\partial r^{*}}\left(\left(r^{*}+R^{*}\right)\frac{\partial T}{\partial r^{*}}\right)+\frac{\mu_{hnl}}{{\left(\rho c_{p}\right)}_{hnl}}{\left(\frac{\partial u_{s^{*}}}{\partial r^{*}}-\frac{u_{s^{*}}}{r^{*}+R^{*}}\right)}^{2},$$

(5) $$u_{s^{*}}=u_{w},\;u_{r^{*}}=0,\;T=T_{w},\;\mathrm{at}\;r^{*}=0,$$

(6) $$u_{s^{*}}\to 0,\;\frac{\partial u_{s^{*}}}{\partial r^{*}}\to 0,\;T\to T_{b},\;\mathrm{as}\;r^{*}\to \infty ,$$

Here, $\left(u_{s^{*}},u_{r^{*}}\right)$ are velocity components in $s^{*}$ and $r^{*}$ directions, p shows $r^{*}$ dependent pressure, T indicates fluid temperature, $T_{w}$ and $T_{b}$ represent the temperature of the curved sheet and bulk fluid, respectively, $\rho_{hnl}$, $k_{hnl}$, ${\left(\rho c_{p}\right)}_{hnl}$ and $\mu_{hnl}$ represent effective density, thermal conductivity, heat capacity and dynamic viscosity of hybrid nanofluids, respectively. The geometry of the flow problem is given below.

### 2.1. Thermophysical Properties 

This section is devoted to presenting the thermophysical properties of convectional and hybrid nanoliquids by either phenomenological law or mixture theory as described below.

#### 2.1.1. Effective Density of Regular and Hybrid Nanoliquid

The effective density of regular nanofluid is given by [34]:

(7) $$\rho_{nl}=\left(1-\phi \right)\rho_{bf}+\phi \rho_{s}.$$

where ϕ represents the solid volume fraction of nanoparticles, $\rho_{bf}$ shows the density of the working fluid and $\rho_{s}$ denotes the density of the nanoparticles. So, the effective density of hybrid nanoliquid $\left(\rho_{hnf}\right)$ is given by:

(8) $$\rho_{hnl}=\phi_{Al_{2}O_{3}}\rho_{Al_{2}O_{3}}+\phi_{Cu}\rho_{Cu}+\left(1-\phi_{Al_{2}O_{3}}-\phi_{Cu}\right)\rho_{bf}.$$

where $\rho_{hnl}$ indicates density of the hybrid nanoliquid, $\rho_{Al_{2}O_{3}}$, $\rho_{Cu}$, $\varphi_{Al_{2}O_{3}}$ and $\phi_{Cu}$ indicate the density of Al_2_O_3_ and Cu nanoparticles and the solid volume fraction of Al_2_O_3_ and Cu nanoparticles, respectively.

#### 2.1.2. Effective Heat Capacitance of Regular and Hybrid Nanoliquids

The effective heat capacitance of convectional nanoliquids ${\left(\rho c_{p}\right)}_{nl}$ is given by:

(9) $${\left(\rho c_{p}\right)}_{nl}=\phi {\left(\rho c_{p}\right)}_{s}+\left(1-\phi \right){\left(\rho c_{p}\right)}_{bf}.$$

Whereas, the effective heat capacitance of hybrid nanoliquids ${\left(\rho c_{p}\right)}_{hnf}$ is given by:

(10) $${\left(\rho c_{p}\right)}_{hnl}=\left(1-\phi_{Al_{2}O_{3}}-\phi_{Cu}\right){\left(\rho c_{p}\right)}_{bf}+\phi_{Al_{2}O_{3}}{\left(\rho c_{p}\right)}_{Al_{2}O_{3}}+\phi_{Cu}{\left(\rho c_{p}\right)}_{Cu}.$$

#### 2.1.3. Effective Thermal Conductivity of Regular and Hybrid Nanoliquids

Based on the Maxwell-Garnetts model [29] the effective thermal conductivity of conventional and hybrid nanoliquids are given, respectively, by:

(11) $$k_{nl}=\frac{\left(k_{s}+2k_{bf}\right)-2\phi \left(k_{bf}-k_{s}\right)}{\left(k_{s}+2k_{bf}\right)+\phi \left(k_{bf}-k_{s}\right)}k_{bf}.$$

(12) $$\frac{k_{hnl}}{k_{bf}}=\frac{\frac{\phi_{Al_{2}O_{3}}k_{Al_{2}O_{3}}+\phi_{Cu}k_{Cu}}{\phi_{Al_{2}O_{3}}+\phi_{Cu}}+2k_{bf}+2\left(\phi_{Al_{2}O_{3}}k_{Al_{2}O_{3}}+\phi_{Cu}k_{Cu}\right)-2\left(\phi_{Al_{2}O_{3}}+\phi_{Cu}\right)k_{bf}}{\frac{\phi_{Al_{2}O_{3}}k_{Al_{2}O_{3}}+\phi_{Cu}k_{Cu}}{\phi_{Al_{2}O_{3}}+\phi_{Cu}}+2k_{bf}-\left(\phi_{Al_{2}O_{3}}k_{Al_{2}O_{3}}+\phi_{Cu}k_{Cu}\right)+\left(\phi_{Al_{2}O_{3}}+\phi_{Cu}\right)k_{bf}}.$$

where the subscripts nl and hnl represent convectional and hybrid nanoliquids, respectively. 

#### 2.1.4. Effective Dynamic Viscosity of Regular and Hybrid Nanoliquids

Based on the Brinkman model [29] the effective dynamic viscosity of regular and hybrid nanoliquids are given, respectively, by:

(13) $$\mu_{nl}=\frac{\mu_{bf}}{{\left(1-\phi \right)}^{2.5}}$$

and:

(14) $$\mu_{hnl}=\frac{\mu_{bf}}{{\left(1-\phi_{Al_{2}O_{3}}-\phi_{Cu}\right)}^{2.5}}$$

Introducing the following dimensionless quantities:

(15) $$ϒ=\sqrt{\frac{u_{w}}{\nu_{bf}}}r^{*},\;F^{\prime }\left(\zeta \right)=\frac{u_{s^{*}}\left(r^{*},s^{*}\right)}{u_{w}},\;u_{r^{*}}\left(r^{*},s^{*}\right)=-\sqrt{u_{o}\nu_{bf}}\frac{R^{*}}{\tilde{r}}F\left(\zeta \right),\;\;T=\theta \left(T_{w}-T_{b}\right)+T_{b},\;p=\rho_{bf}u_{0}^{2}s^{*}{}^{2}P\left(\zeta \right),\;\kappa =R^{*}\sqrt{\frac{u_{o}}{\nu^{*}}}\;(\mathrm{dimensionless}\;\mathrm{radius}\;\;\mathrm{of}\;\mathrm{curvature}),\;Ec=\frac{u_{w}^{2}}{{\left(c_{p}\right)}_{bf}\left(T_{w}-T_{b}\right)}\;(\mathrm{Eckert}\;\mathrm{number})\;\mathrm{and}\;\mathrm{Pr}={\left(\frac{\nu }{\alpha }\right)}_{bf}\;\;(\mathrm{Prandtl}\;\mathrm{number}).$$

into Equations (2)–(6), we get:

(16) $$\frac{\partial P}{\partial \zeta }=H_{o}\frac{{F^{\prime }}^{2}}{\zeta +\kappa }$$

(17) $$2\frac{P\kappa }{H_{o}\left(\kappa +\zeta \right)}=\frac{1}{H_{o}H_{1}}\left(F^{\prime \prime \prime }+\frac{F^{\prime \prime }}{\kappa +\zeta }-\frac{F^{\prime }}{{\left(\kappa +\zeta \right)}^{2}}\right)+\frac{\kappa }{\kappa +\zeta }FF^{\prime \prime }+\frac{\kappa }{{\left(\kappa +\zeta \right)}^{2}}FF^{\prime }-\frac{\kappa }{\kappa +\zeta }{F^{\prime }}^{2},$$

(18) $$\frac{H_{3}}{H_{2}\mathrm{Pr}}\left(\frac{\theta^{\prime }}{\kappa +\zeta }+\theta^{\prime \prime }\right)+\frac{\kappa }{\kappa +\zeta }\left(F\theta^{\prime }-2F^{\prime }\theta \right)+\frac{Ec}{H_{1}H_{2}}{\left(F^{\prime \prime }-\frac{F^{\prime }}{\kappa +\zeta }\right)}^{2}=0,$$

(19) $$F\left(0\right)=0,\;F^{\prime }\left(0\right)=1,\;\theta \left(0\right)=1,$$

(20) $$F^{\prime }\left(\zeta \to \infty \right)\to 0,\;F\prime \prime \left(\zeta \to \infty \right)\to 0,\;\theta \left(\zeta \to \infty \right)\to 0.$$

Eliminating dimensionless pressure P from Equation (8) by using Equation (7), we get:

(21) $$F\prime \prime \prime \prime +\frac{2}{\left(\kappa +\zeta \right)}F^{\prime \prime \prime }-\frac{F^{\prime \prime }}{{\left(\kappa +\zeta \right)}^{2}}+\frac{F^{\prime }}{{\left(\kappa +\zeta \right)}^{3}}+\kappa H_{o}H_{1}\left(-\frac{F^{\prime }F^{\prime \prime }}{\left(\kappa +\zeta \right)}+\frac{FF^{\prime \prime \prime }}{\left(\kappa +\zeta \right)}-\frac{{F^{\prime }}^{2}}{{\left(\kappa +\zeta \right)}^{2}}+\frac{FF^{\prime \prime }}{{\left(\kappa +\zeta \right)}^{2}}-\frac{FF^{\prime }}{{\left(\kappa +\zeta \right)}^{3}}\right)=0.$$

Here:

(22) $$H_{o}=\left(1-\phi_{Al_{2}O_{3}}-\phi_{Cu}+\frac{\rho_{Al_{2}O_{3}}\phi_{Al_{2}O_{3}}+\rho_{Cu}\phi_{Cu}}{\rho_{bf}}\right),\;H_{1}={\left(1-\phi_{Cu}-\phi_{Al_{2}O_{3}}\right)}^{5/2},H_{3}=\frac{k_{hnl}}{k_{bf}},\;H_{2}=\left(1-\phi_{Al_{2}O_{3}}-\phi_{Cu}+\frac{{\left(\phi \rho c_{p}\right)}_{Al_{2}O_{3}}+{\left(\phi \rho c_{p}\right)}_{{}_{cu}}}{{\left(\rho c_{p}\right)}_{bf}}\right),$$

## 3. Entropy Generation Minimization (EGM)

The expression of entropy generation for an incompressible hybrid nanofluid flow over an elastic curved surface in the presence of energy dissipation takes the following form [35]:

(23) $${{\dot{E}}^{\prime \prime \prime }}_{Gen}=\frac{k_{hnl}}{T^{2}}{\left(\frac{\partial T}{\partial r^{*}}\right)}^{2}+\frac{\mu_{hnl}}{T}{\left(\frac{\partial u_{s^{*}}}{\partial r^{*}}-\frac{u_{s^{*}}}{r^{*}+R^{*}}\right)}^{2}$$

The first term $\frac{k_{hnl}}{T^{2}}{\left(\frac{\partial T}{\partial r^{*}}\right)}^{2}$ on the right-hand side of Equation (23) shows the entropy production by heat transfer and the last term $\frac{\mu_{hnl}}{T}{\left(\frac{\partial u_{s^{*}}}{\partial r^{*}}-\frac{u_{s^{*}}}{r^{*}+R^{*}}\right)}^{2}$ represents the entropy production by energy dissipation.

Substituting Equation (7) into Equation (23), we get the dimensionless form of entropy production Ns as given below:

(24) $$N_{s}={\frac{{{\dot{E}}^{\prime \prime \prime }}_{Gen}}{\left({{\dot{E}}^{\prime \prime \prime }}_{Gen}\right)}}_{c}=\underset{Thermal\;\mathrm{irreversibility}}{\underset{︸}{\frac{H_{3}}{{\left(\theta +\lambda \right)}^{2}}{\theta^{\prime }}^{2}}}+\underset{Frictional\;irreversibility}{\underset{︸}{\frac{Ec\mathrm{Pr}{\left(g^{\prime \prime }-\frac{g^{\prime }}{\kappa +\zeta }\right)}^{2}}{H_{1}\left(\theta +\lambda \right)}}}.$$

Here,${\left({{\dot{E}}^{\prime \prime \prime }}_{Gen}\right)}_{o}={\left(\frac{k}{\nu }\right)}_{bf}u_{o}$ (characteristic entropy generation) and $\lambda =\frac{T_{b}}{T_{w}-T_{b}}$ (temperature difference parameter).

## 4. Results and Discussion 

The core objective of the present section is to discuss the impacts of different dimensionless parameters such as the Eckert number Ec, the nanoparticle solid volume fraction ϕ, the curvature parameter κ and the temperature difference parameter λ. Since water has been used as a working fluid, the Prandtl number Pr = 6.8 has been used. Table 1 represents the thermophysical properties of the working fluid (water) and nanoparticles (copper and aluminium oxide). The boundary layer flow driven by a curved surface is a complex two-point boundary value problem. The governing set of Equations (18) and (21) are highly non-linear. The closed-form solutions of the previously mentioned equations are not possible. Therefore, we adopted the Runge-Kutta method along with the shooting method for solving these non-linear equations. To ensure the correctness of the present results, the system of Equations (18) and (21) are also solved by using the Matlab in-built boundary value solver, i.e., bvp4c. Table 2 shows an excellent agreement among the numerical values of the local skin friction coefficient in the absences of nanoparticles $\left(\phi_{Cu}=\phi_{Al_{2}O_{3}}=0\right)$ for different values of dimensionless radius of curvature (κ). This is the first validation test of our numerical results. A comparison with the work of Pop and Rosca [34] has been made as shown in Table 3. Table 3 is made by taking $\phi_{Cu}=\phi_{Al_{2}O_{3}}=Ec=0$ and different values of κ. Table 3 shows that numerical values of the local skin friction coefficient −Res*0.5Cfs* computed by [34] and the present results are in good agreement and hence validate our present results. This is the second test for validation of the present numerical simulation. 

**Figure 2:**
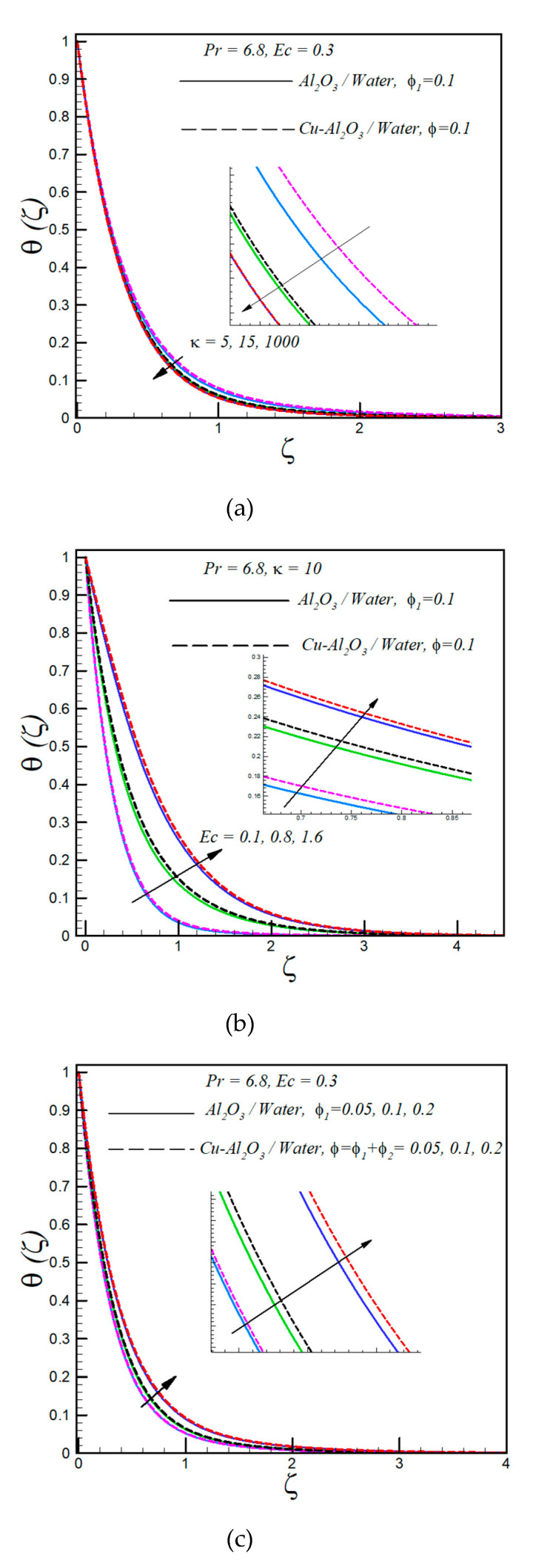
(**a**) Variation of temperature profile θ(ζ) with dimensionless radius of curvature (**b**) Eckert number and (**c**) solid volume fraction.

The change in temperature profile with increasing values of the curvature parameter κ is shown in Figure 2a. As presented, with an increase in κ (decreasing curvature of the stretching surface) the temperature decreases. Besides, it is found that hybrid nanofluid has a slightly higher temperature than regular nanofluid; this is because of the high thermal conductivity of hybrid nanofluid. Further, for fixed values of κ, the temperature profile decreases asymptotically with increasing values of ζ. Figure 2b displays the influence of varying the viscous dissipation parameter Ec (Eckert number) on the temperature distribution of both hybrid/regular nanofluids. A significant rise in temperature is observed with increasing values of Ec. This increment in temperature with an increasing Eckert number is because of frictional heating. Besides, the temperature of regular nanofluid is low as compared to hybrid nanofluid due to low thermal conductivity. The asymptotic decrement of the temperature profile with increasing values of the similarity variable is also shown in Figure 2b. Figure 2b also shows that the thermal boundary layer is thick in cases of flow over a curved surface as compared to flow over a flat surface. The plot of temperature against various values of the solid volume fraction is shown in Figure 2c. The boosts in the temperature profile for both types of hybrid/regular nanofluids is found with increasing values of the solid volume fraction. According to the Hamilton and Crosser model, the thermal conductivity of nanofluid increases with increasing values of the solid volume fraction and this increment in thermal conductivity leads to an enhancement of the rate of heat transfer. For fixed values of the solid volume fraction, an asymptotic decrement is observed in temperature profiles for both types of nanofluids.

**Figure 3:**
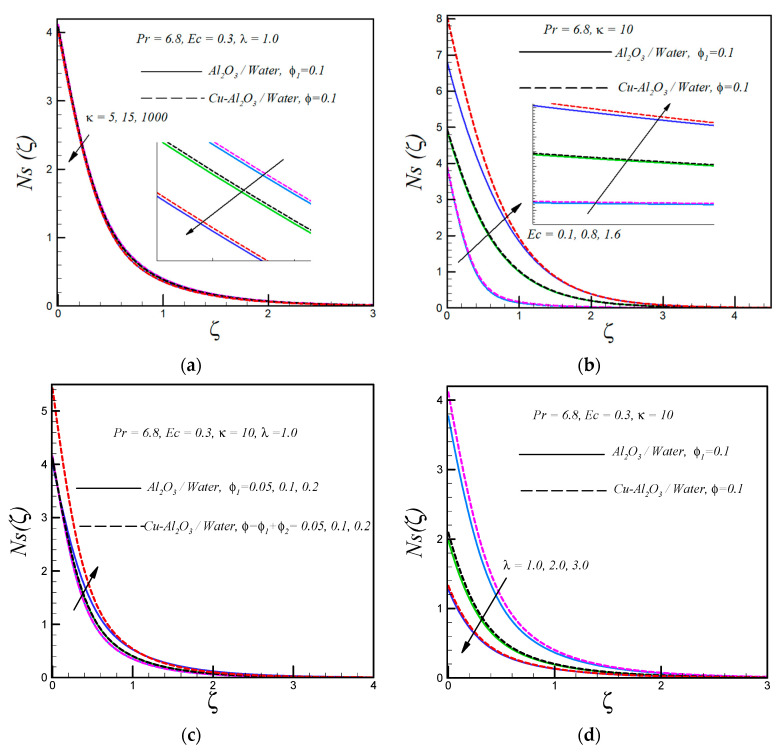
(**a**) Variation of entropy generation number Ns(ζ) with dimensionless radius of curvature, (**b**) Eckert number, (**c**) solid volume fraction and (**d**) temperature difference parameter.

The distribution of entropy generation for various values of the curvature parameter κ, keeping all the other parameters constant, is shown in Figure 3a. This plot shows that entropy generation reduces with an increase in κ, i.e., a decrease in the curvature of the curved surface. Moreover, the rate of entropy generation is low in nanofluids containing Cu nanoparticles as compared to nanofluids containing Cu-Al_2_O_3_ nanoparticles. It is also noticed that entropy generation is less in cases of flow over a flat surface as compared to the flow over a curved surface. An increase in the viscous dissipation parameter (Eckert number) leads to an enhancement of the rate of entropy generation for both types of nanofluids, as depicted in Figure 3b. Physically, entropy generation Ns is an increasing function of non-conservative forces (such as frictional forces). Therefore, the Ns profile boosts with an increasing Eckert number (increasing frictional forces between the fluid layers). It is a well-known fact that the rate of heat transfer increases with increasing values of the solid volume fraction and consequently the rate of entropy generation rises with rising values of the solid volume fraction, as shown in Figure 3c. Further, it is also noticeable that the entropy generation is high at the surface of the curve boundary due to high velocity and thermal gradients. An increase in the temperature difference parameter λ (decreasing temperature difference between the curved surface and ambient fluid) reduces the entropy generation. This can be explained as the rate of heat transfer reduces with reduction in the operating temperature ΔT and consequently the rate of entropy generation decreases. Further, it is observed that Ns is high in nanofluids containing hybrid nanoparticles as compare to regular nanofluids. 

## 5. Concluding Remarks

In the present work, entropy generation in the dissipative flow of conventional nanofluid and hybrid nanofluid are investigated. The flow is driven by an elastic curved surface. The effects of different emerging parameters on the temperature profile are also investigated here. Based on the present investigation, the following observations are made:

- An increment in the dimensionless radius of curvature leads to suppression of the temperature profile.
- An increment in the Eckert number and solid volume fraction of nanoparticles leads to boosting the temperature profile.
- A high rate of heat transfer is observed in hybrid nanofluid as compare to regular nanofluid.
- The entropy generation profile is suppressed by rising values of the temperature difference parameter.
- Under the same circumstances, the rate of entropy generation is less in a flow over a flat surface as compared to the curved surface.
- The entropy generation Ns enhances with rising values of the solid volume fraction and the Eckert number.
- Less entropy is generated in the flow of regular nanofluid as compare to hybrid nanofluid.
- The entropy generation Ns is maximum at the solid boundary.

## Figures and Tables

**Table 1:** Thermophysical properties of base fluid and some nanoparticles.

Properties	Base Fluid (Water)	Al_2_O_3_ (Aluminum Oxide)	Cu (Copper)
$c_{p}\left(J/kgK\right)$	4179	765	385
k(W/mK)	0.613	40	401
$\rho \left(kg/m^{3}\right)$	997.1	3970	8933
Pr	6.8	-	-

**Table 2:** Comparison of numerical results for −Res*0.5Cfs*, when $\phi_{Cu}=\phi_{Al_{2}O_{3}}=Ec=0$.

κ	Present Numerical Results	
	Bvp4c	RKFM
5	1.157632	1.157631
10	1.073487	1.073488
20	1.035610	1.035609
30	1.023531	1.023531
40	1.017588	1.017586
50	1.014048	1.014049
100	1.007039	1.007038
200	1.003554	1.003564
1000	1.000798	1.000799

**Table 3:** Comparison of our numerical values for −Res*0.5Cfs* with existing results for different values of κ, when $\phi_{Cu}=\phi_{Al_{2}O_{3}}=Ec=0$.

κ	Rosca and Pop [36]	Present Results
5	1.15076	1.1576312
10	1.07172	1.0734886
20	1.03501	1.0356098
30	1.02315	1.0235310
40	1.01729	1.0175866
50	1.01380	1.0140492
100	1.00687	1.0070384
200	1.00342	1.0035641
1000	1.00068	1.0007993
